# ﻿Two new species of *Varicus* from Caribbean deep reefs, with comments on the related genus *Pinnichthys* (Teleostei, Gobiidae, Gobiosomatini, Nes subgroup)

**DOI:** 10.3897/zookeys.1180.107551

**Published:** 2023-09-20

**Authors:** Katlyn M. Fuentes, Carole C. Baldwin, D. Ross Robertson, Claudia C. Lardizábal, Luke Tornabene

**Affiliations:** 1 School of Aquatic and Fishery Sciences and the Burke Museum of Natural History and Culture, University of Washington, 1122 NE Boat Street, Seattle, WA 98105, USA University of Washington Seattle United States of America; 2 Department of Vertebrate Zoology, National Museum of Natural History, Smithsonian Institution, PO Box 37012, Washington, DC 20013-7012, USA National Museum of Natural History, Smithsonian Institution Washington, DC United States of America; 3 Smithsonian Tropical Research Institute, Balboa, Panama Smithsonian Tropical Research Institute Balboa Panama; 4 Instituto de Investigación en Ciencias Biológicas y Ambientales del Norte de Honduras (IBIOANH), Departamento de Biología, Universidad Nacional Autónoma de Honduras en el Valle de Sula, Zona el Playon, final del Blvd Micheletti, San Pedro Sula 21102, Cortes, Honduras Universidad Nacional Autónoma de Honduras en el Valle de Sula San Pedro Sula Honduras

**Keywords:** Gobies, ichthyology, mesophotic, molecular systematics, phylogenetics, rariphotic, submersible

## Abstract

Tropical deep reefs (~40–300 m) are diverse ecosystems that serve as habitats for diverse communities of reef-associated fishes. Deep-reef fish communities are taxonomically and ecologically distinct from those on shallow reefs, but like those on shallow reefs, they are home to a species-rich assemblage of small, cryptobenthic reef fishes, including many species from the family Gobiidae (gobies). Here we describe two new species of deep-reef gobies, *Varicusprometheus***sp. nov.** and *V.roatanensis***sp. nov.**, that were collected using the submersible *Idabel* from rariphotic reefs off the island of Roatan (Honduras) in the Caribbean. The new species are the 11^th^ and 12^th^ species of the genus *Varicus*, and their placement in the genus is supported by morphological data and molecular phylogenetic analyses. Additionally, we also collected new specimens of the closely-related genus and species *Pinnichthysaimoriensis* during submersible collections off the islands of Bonaire and St. Eustatius (Netherland Antilles) and included them in this study to expand the current description of that species and document its range extension from Brazil into the Caribbean. Collectively, the two new species of *Varicus* and new records of *P.aimoriensis* add to our growing knowledge of cryptobenthic fish diversity on deep reefs of the Caribbean.

## ﻿Introduction

Deep reefs occur worldwide in tropical and subtropical regions at depths ranging from approximately 40 m to at least 300 m (Baldwin et al. 2018). Despite their global distribution, our knowledge of fish communities on deep reefs is limited when compared to those on shallow reefs, as the depths require the use of submersibles, remote operated vehicles (ROVs), baited underwater cameras, or closed-circuit rebreathers. However, the use of human-occupied submersibles, especially within the last decade, has greatly expanded our knowledge of Caribbean fish communities on mesophotic (~40–130 m) and rariphotic (~130–300 m) ecosystems (Baldwin et al. 2018; Robertson et al. 2020, 2022).

Presently, there is not only a lack of studies focused on fish communities spanning the entire depth range of deep reefs, but also on cryptobenthic reef fishes that reside in these ecosystems (Robertson et al. 2020, 2022). Cryptobenthic reef fishes are small species (typically < 50 mm total length) that live on or near the seafloor, take refuge in crevices or by camouflaging themselves, and are often overlooked during visual surveys (Brandl et al. 2018). Cryptobenthic reef fishes account for a substantial fraction (50% or more at some sites) of the overall diversity of reef fishes on both shallow and deep reefs and contribute heavily to the marine trophic web (Depczynski and Bellwood 2003; Brandl et al. 2018, 2019; Robertson and Tornabene 2020; Robertson et al. 2022). Although cryptobenthic reef fishes are typically understudied on shallow reefs, this knowledge gap is even greater on deep reefs, especially at sites that have not had submersible-based collections (Robertson et al. 2022).

One group of cryptobenthic reef fishes that are well-represented throughout shallow and deep reefs, are gobies (Gobiidae). Gobies are among the most diverse families of marine fishes, and with more than 160 known species in the Greater Caribbean, are the most diverse family of shorefishes in that region. These fishes exhibit a variety of morphological and behavioral adaptations that have allowed them to thrive in aquatic ecosystems worldwide (Patzner et al. 2011). In the Caribbean, new species of Gobiidae have recently been described as a result of submersible collections led by the Smithsonian Deep Reef Observation Project (DROP; Baldwin and Robertson 2015; Tornabene et al. 2016a, b, c; Tornabene and Baldwin 2017; Tornabene and Baldwin 2019; Tornabene et al. 2022). One of the first DROP studies on gobies described nine new species and erected four new genera (Tornabene et al. 2016b). Included in that study were five new species in the genus *Varicus* Robins & Böhlke, 1961, which is the most diverse genus of deep-reef gobies in the Caribbean (10 species prior to this study). That study also erected the genus *Pinnichthys* Van Tassell, Tornabene & Gilmore, 2016 for the type species *Pinnichthysaimoriensis* Van Tassell & Tornabene, 2016, which was found at 70 m off the coast of Espírito Santo, Brazil, and four additional species from the tropical western Atlantic and tropical eastern Pacific (Tornabene et al. 2016b). In 2017 and 2018, DROP expanded research to Bonaire, St. Eustatius and Roatan. These trips substantially increased our knowledge of deep-reef fishes from these islands (Robertson et al. 2020, 2022) and resulted in the discovery of additional undescribed species (Tornabene et al. 2018; Tornabene and Baldwin 2019; Tornabene et al. 2022), as well as the collection of additional specimens of *P.aimoriensis*.

Here we describe two new species representing the 11^th^ and 12^th^ species of the genus *Varicus* based on two specimens collected in 2018 from Roatan, Honduras. Additionally, we provide comments to expand the current species description of *P.aimoriensis* based on two specimens collected in 2017 by DROP from Bonaire and Sint Eustatius, as well as updated scale counts from the type specimens. The two new specimens are nearly 6000 km from the type locality of *P.aimoriensis* and were collected at greater depths than the original types (96 m and 164 m versus 70 m).

## ﻿Material and method

The new species of *Varicus* (UW 158119 and UW 158127) were collected off the coast of Half Moon Bay, Roatan, Honduras, using the *Idabel* submersible, which accommodates three people (one pilot and two passengers) and has a maximum depth rating of 915 m. Prior to capture the specimens were sedated with a solution of quinaldine-sulfate in sea water, which was dispersed via a pump through a tube on the front of the submersible. Sedated specimens were then collected by a suction hose powered by one of the sub’s vertical thrusters and stored in an acrylic collection tank attached to the sub until they were brought to the surface (see Tornabene et al. 2018 for an image of the collection apparatus). Fishes were photographed in a water-filled phototank and tissue-sampled prior to fixation in 10% formalin and subsequent storage in 70% ETOH. Both holotypes are deposited at the University of Washington Burke Museum Fish Collection, Seattle. The holotype of one of the new species (*Varicusprometheus* sp. nov.; UW 158119) was partially damaged after collection by a pagurid crustacean that was collected on the same dive and stored in the collection tank. Therefore, the measurement of certain morphological characters (i.e., depth at origin of 1^st^ dorsal fin) were approximated.

Two specimens of *Pinnichthys* (USNM 442071 and USNM 442696) were collected via the *Curasub* submersible off the coasts of Bonaire and St. Eustatius. Additional *P.aimoriensis* paratypes (AMNH 265021, AMNH 265020) were also examined. Discrepancies in the number of transverse scale rows between the type specimens and the original description were noted and are corrected here (see Table 2).

All measurements were taken using digital calipers to the nearest 0.1 mm, and all morphological characters analyzed here are defined by Böhlke and Robins (1968) as modified by Van Tassell et al. (2012). These characters were taken for all specimens listed above and compared to counts and measurements for all species of *Pinnichthys* and *Varicus*. Digital radiographs of several specimens, including type specimens, were taken to get accurate counts of fin rays, vertebrae, and other osteological features. The holotypes of the new species were temporarily stained with cyanine blue following Saruwatari et al. (1997) to help visualize sensory neuromast (papillae) patterns on the head. Sensory papillae are described according to Sanzo (1911), with modifications according to Tornabene et al. (2016b).

Methods for DNA analysis follow those of Tornabene et al. (2016b). DNA was extracted from tissue samples using a Qiagen DNeasy Blood and Tissue Kit. To confirm the phylogenetic placement of the four specimens (two new species of *Varicus* and two specimens of *Pinnichthysaimoriensis*), the mitochondrial gene cytochrome *b* and three nuclear genes (Rag1, Sreb2, and Zic1) were amplified via polymerase chain reaction (PCR) using primers and protocols described in Agorreta et al. (2013). Amplification success varied across the samples (Appendix 1). PCR results were then visualized by gel electrophoresis and sequenced by Molecular Cloning Laboratories (MCLAB, South San Francisco, California). Sequence data from these samples were assembled and aligned in Geneious Prime version 2022.2.2 (https://www.geneious.com, accessed 30 Aug 2022), and combined with alignments from previous studies (Tornabene et al. 2016a, b, 2022; Tornabene and Baldwin 2019). The final matrix included 119 taxa, with 51 taxa from the *Nes* subgroup of the tribe Gobiosomatini, of which *Pinnichthys* and *Varicus* are members. A phylogeny was inferred from the 4389 base-pair concatenated alignment using Bayesian inference. The best-fitting partitioning scheme and evolutionary model choice were determined using PartitionFinder2 (Lanfear et al. 2016). A Metropolis-coupled MCMC was run for 10 million generations sampling every 1000 generations in the software MrBayes version 3.2 (Ronquist et al. 2012). Mixing and convergence were assessed using TRACER (Rambaut and Drummond 2007) and the resulting 50% majority rule consensus tree was visualized using FigTree (http://tree.bio.ed.ac.uk/software/figtree/, accessed 30 August 2022). New sequences generated here are deposited in GenBank (Appendix 1).

## ﻿Results

**Figure 1.**
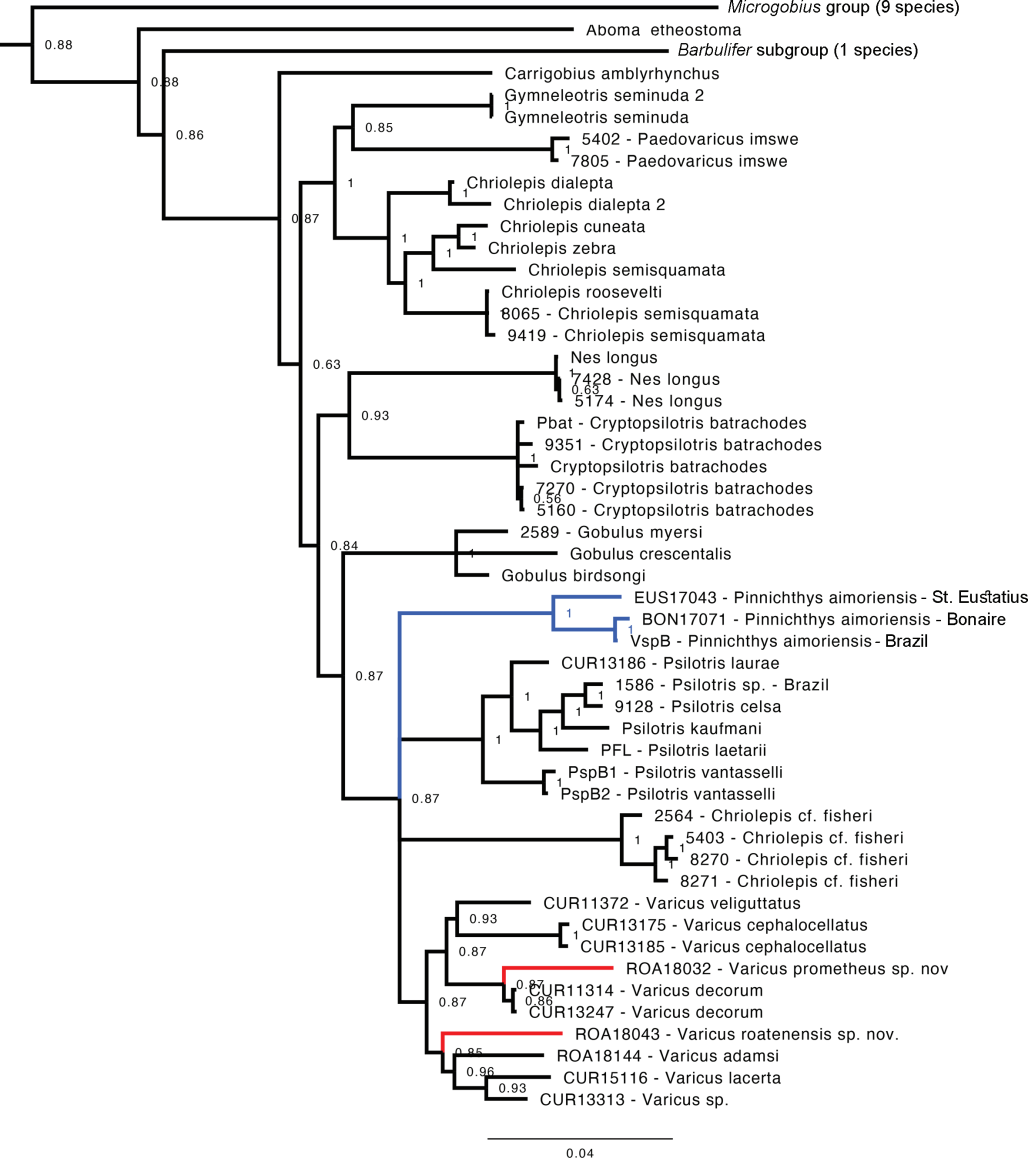
Molecular phylogeny of Gobiosomatini based on three nuclear genes and one mitochondrial gene. Support values are Bayesian posterior probabilities. Full tree of Gobiidae available as Supporting Information.

The molecular phylogenetic analysis shows strong support for monophyly of the tribe Gobiosomatini, the *Gobiosoma* group, and the *Nes* subgroup (Fig. 1; see Suppl. material 1: fig. S1 for full 119 taxa tree). The genus *Varicus* is recovered as monophyletic with moderately strong support (posterior probability = 0.87), with the relationships between *Varicus*, *Pinnichthys*, *Psilotris*, and Chriolepiscf.fisheri being unresolved. The two new species from Roatan (UW 158127, UW 158119) are recovered within the genus *Varicus*. *Varicusroatanensis* sp. nov., is sister to a clade containing *V.adamsi*, *V.lacerta*, and an undescribed species of *Varicus* from Curaçao. *Varicusprometheus* sp. nov., UW 158119 is sister to *V.decorum*. Relationships of both new species within the genus are supported with moderately strong posterior probabilities of 0.85–0.87.

All three specimens of *Pinnichthysaimoriensis* are recovered as a monophyletic group with strong support (posterior probability = 1.0). The specimen from Bonaire is most closely related to the holotype from Brazil, with the specimen from St. Eustatius being sister to them on a comparatively longer branch. However, we were only successful in amplifying partial segments of two out of four genes and (1551 out of 4389 bp, or 64.6% missing data), so we are cautious about our interpretation of the exact position and branch lengths for this specimen.

### ﻿Taxonomy

#### ﻿ Varicus prometheus sp. nov.

Taxon classificationAnimaliaPerciformesGobiidae

68988650-4CC2-5D18-ABB4-FFFD6CE13EBB

https://zoobank.org/E996CE37-FF77-4BA4-A550-039380D8ABBE

**Figure 2.**
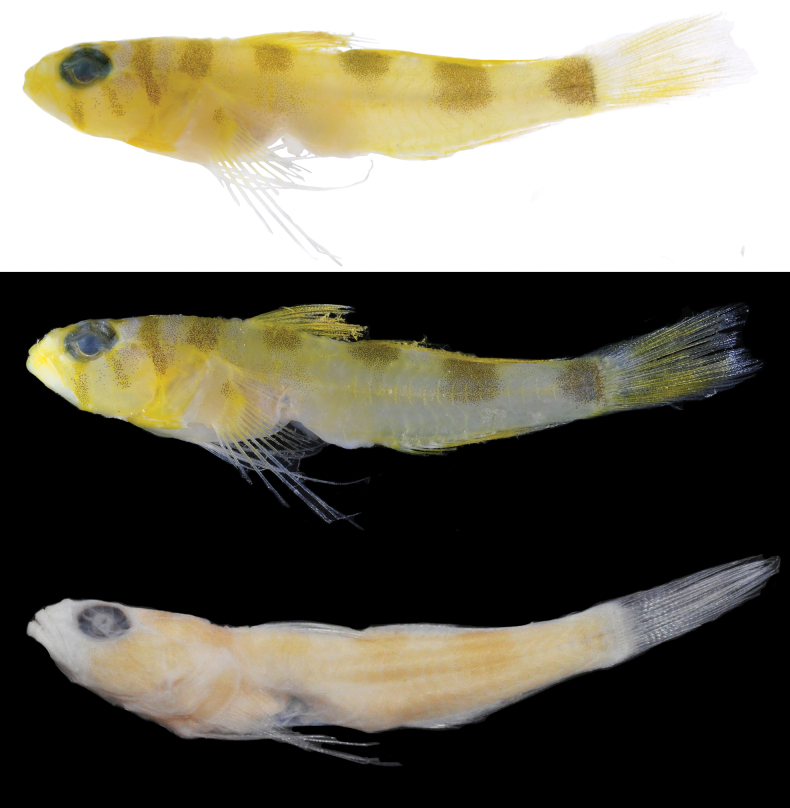
*Varicusprometheus* sp. nov. holotype, UW 158119, 30.5 mm SL, female, prior to preservation (top two photos) and preserved (bottom). Photos by Luke Tornabene.

**Figure 3.**
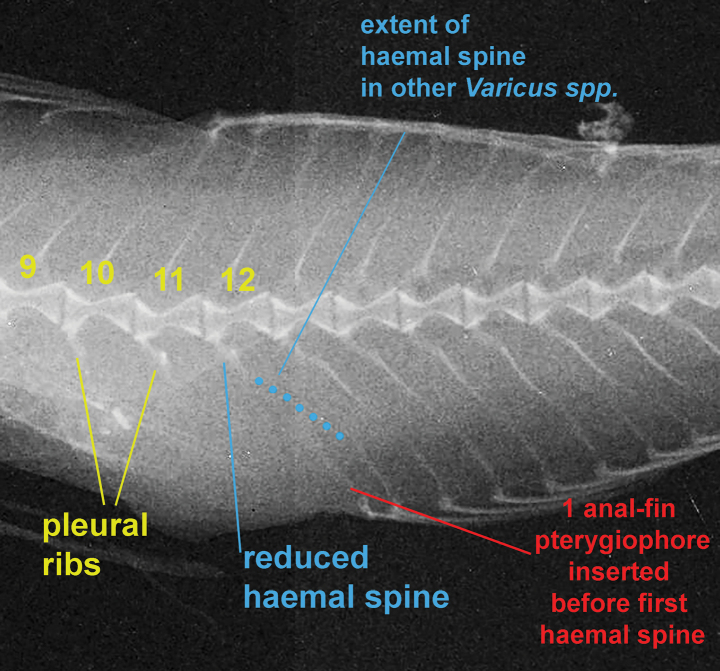
Vertebral skeleton and anal-fin pterygiophores of *Varicusprometheus* sp. nov. Yellow numbers are the number of vertebrae.

**Figure 4.**
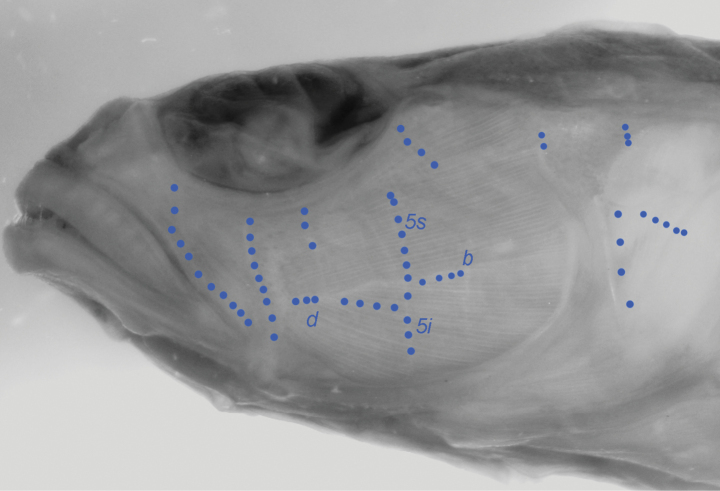
Sensory papillae pattern for *Varicusprometheus*. Labels for papillae rows follow Sanzo (1911) as modified by Tornabene et al. (2016b). Photo by L. Tornabene.

Figs 2 , 3 , 4 Promethean goby, Gobio prometeico (Spanish)

##### Type locality.

Roatan, Honduras; western Caribbean.

##### Holotype.

Honduras • 1 female 30.5 mm SL; western Caribbean, island of Roatan, west End, off Half Moon Bay, sta. IDABEL18-02; 16.304°N, 86.598°W; 247 m depth; 5 June 2018; Luke Tornabene, D. Ross Robertson and Karl Stanley; 5% quinaldine-sulfate dispersed from Idabel submersible; UW 158119, DNA sample ROA18032.

##### Generic placement.

In addition to molecular characters supporting the phylogenetic placement of this species, the following morphological characters support its inclusion in the genus *Varicus* (*sensu*Tornabene et al. 2016b): dorsal-fin pterygiophore formula of 3-221110; 27 vertebrae (11 precaudal + 16 caudal, but see “Remarks” section below); one anal-fin pterygiophore inserted anterior to first haemal spine (but see ‘Remarks’ section below); anal-fin rays I,8; head pores absent; pelvic fins completely separate, lacking both anterior frenum and membrane connecting bases of innermost pelvic-fin rays; fifth pelvic-fin ray unbranched.

##### Diagnosis.

*Varicusprometheus* is distinguishable from all other *Varicus* species by the following combination of characters: second dorsal fin I,9; anal fin I,8; pectoral fin 17; scales absent except for two modified basicaudal scales; pelvic rays 1–4 unbranched without fleshy tips; one anal-fin pterygiophore inserted anterior to first haemal spine (but see ‘Remarks’ section below); body with four incomplete brown saddles on pale to bright yellow background; pelvic and pectoral fins white to pale yellow in life, dorsal, anal, and caudal fins pale to bright yellow.

##### Description.

***General shape***: body robust, widest and deepest at posterior of head, trunk tapering in width and depth posteriorly, dorsal profile of head gently sloping from dorsum to tip of snout.

***Morphometrics (%SL)***: head length 26.9; eye diameter 7.5; snout length 7.5; upper-jaw length 12.8; post-orbital length 13.1; predorsal length 36.7; body depth at 1^st^ dorsal-fin origin 18.7; body depth at anal-fin origin 17.0; preanal length 61.0; body depth at caudal peduncle 10.5; caudal-peduncle length 12.1; pectoral-fin length 32.8; pelvic-fin length 25.6.

***Median and paired fins***: first dorsal fin VII; second dorsal fin I,9; anal fin I,8; pectoral fin 17; pelvic fins I,5, fins well separated, lacking both anterior frenum and membrane connecting bases of innermost rays; 4^th^ pelvic-fin ray longest, extending posteriorly to base of anal-fin spine; rays 1–4 all unbranched, no fleshy tips; 5^th^ ray unbranched and <10% the length of the 4^th^ ray; caudal fin shape undeterminable due to poor condition of specimen, branched caudal-fin rays 13, segmented caudal-fin rays 17.

***Vertebral skeleton* (Fig. 3)**: 27 vertebrae, 11 precaudal vertebrae, 16 caudal vertebrae (see ‘Remarks’ section below); dorsal-fin pterygiophore insertion pattern of 3-221110.

***Head***: jaw terminal, angled approximately 45 degrees from horizontal axis of the body, extending posteriorly to vertical through anterior margin of pupil; anterior nares on a short tube, posterior nares small, barely visible as an opening on a slightly raised rim immediately anterior to eye; eyes large, dorsolateral, extending to dorsal profile of head; operculum opening slightly wider than width of pectoral-fin base; teeth difficult to accurately assess due to condition of specimen, teeth in both upper and lower jaws in multiple rows, two to three rows anteriorly, with outer row in upper jaw slightly enlarged with recurved canines.

***Sensory papillae* (Fig. 4)**: sensory papillae on the sides of head of holotype are badly damaged, and the following description is a composite of both sides of the head. Five transverse rows extending from below eye, with two longitudinal rows; first longitudinal row (row ‘d’ of Sanzo 1911) at level just above angle of jaw row, extending between second and fourth transverse row; second longitudinal row (row ‘b’ of Sanzo 1911) short, originating below posterior margin of eye immediately posterior to fourth transverse row; first transverse row originating at anteroventral corner of eye, second transverse row below originating at anterior margin of pupil, third transverse row originating below posterior margin of pupil, fourth transverse row (row 5i/5s of Tornabene et al. 2016b) originating below posterior margin of eye and extending below longitudinal as a single continuous row (5i/5s continuous versus separate), fifth transverse row diagonal, originating at posterior margin of eye, well above fourth row; several short vertical rows of papillae above operculum; a vertical row of four evenly space papillae near anterior margin of operculum; a short oblique row of papillae across middle of operculum; papillae row p not apparent except for a single papilla peʹ behind each eye; body with approximately 15 short vertical rows of papillae evenly spaced along lateral midline, beginning below origin of second dorsal fin and continuing onto caudal peduncle.

***Squamation***: scales on head and body absent except for two modified basicaudal scales with enlarged ctenii present at dorsal and ventral margins of caudal-fin base.

***Genitalia***: female with short, slightly rounded, conical papilla, wider at base and tapering to a slight point; male unknown.

***Color before preservation* (Fig. 2)**: background color of head and body white to pale yellow, with upper portion of head and body slightly brighter yellow than posterior half of body (pigmentation of which is more visible when fish was photographed against white vs. black background). Head with areas of bright yellow pigment lightly speckled with small brown melanophores across upper jaw and snout; four dark bars formed by speckled brown melanophores; first two bars, slightly narrower than pupil diameter, extending ventrally from interorbital, one across the anterior eye and ending at posterior tip of jaw and the second crossing slightly posterior to pupil and ending mid-cheek; third bar originating well behind eye at dorsal midline and extending ventrally slightly onto middle of opercle; and fourth bar wider than first three, anterior to first dorsal fin, extending ventrally to approximately to just above pectoral-fin base. Eyes with green-yellow irises speckled with silvery dots and a thin silvery ring around the pupil. Body pale yellow to white with four dark saddles heavily speckled with brown melanophores; first saddle slightly narrower in width than eye diameter, located at middle of base of first dorsal fin extending ventrally to lateral midline where melanophores become less dense; second saddle about equal in width to eye diameter, located at anterior of second dorsal fin extending ventrally just short of lateral midline; third saddle slightly wider than eye diameter, located under rear of second dorsal fin base, extending ventrally to just beyond lateral midline; fourth saddle located laterally on the caudal peduncle at base of caudal fin, the front border rounded and the rear border straight, slightly larger than eye diameter. First dorsal fin bright yellow with a few brown melanophores along base immediately above saddle on body, few scattered melanophores on interradial membranes; second dorsal fin similarly colored as first. Caudal fin with a very wide oblique yellow bar down middle of fin rays. Anal fin bright yellow. Pectoral-fin base yellow with blotch of brown melanophores at center on the upper third of the fin base, extending to origin of rays. Pelvic fins pale.

***Color in preservation* (Fig. 2)**: body and head uniformly pale yellow, fins translucent. Only remaining pigment consisting of light speckling of small melanophores on trunk at location where dark saddles occurred in life.

##### Habitat.

The holotype was collected at from the deep-reef slope at 247 m on sand with *Halimeda* rubble.

##### Distribution.

Known only from the type locality off Roatan, Honduras.

##### Etymology.

The specific epithet *prometheus* is a patronym in reference to the Greek god Prometheus. In Greek mythology, as punishment from the god Zeus, Prometheus had his liver eaten out by an eagle, only to have the liver grow back overnight so it might be eaten again the next day. The name refers to the fact that the abdomen of the holotype of the new species was partially eaten by a hermit crab. The name is treated as a noun in apposition.

##### Remarks.

*Varicusprometheus* most likely possesses 27 total vertebrae, like all other species in genus. However, our radiographs did not have enough resolution for us to determine whether the 12^th^ vertebra had a haemal spine (if present, it is very reduced) rendering it the first caudal vertebra versus the last precaudal vertebra (Fig. 3). Thus, the counts of precaudal and caudal vertebrae may be 11+17 or 12+16. Accordingly, it is unclear how many anal-fin pterygiophores are inserted anterior to the first haemal spine. All other species of *Varicus* have one anal-fin pterygiophore inserted anterior to the haemal arch that is present on the 12^th^ vertebra (other species of Gobiosomatini have two pterygiophores inserted here, including the morphologically similar genus *Psilotris* Ginsburg, 1953). In some rare instances in *Varicus*, the haemal arch on the 12^th^ vertebra is very reduced and does not insert deeply into the space between the first two anal-fin pterygiophores, making it incorrectly appear on the x-ray as if there are two anal pterygiophores inserted before the “first” haemal spine on the 13^th^ vertebrae, rather than one pterygiophore inserted before the 12^th^ vertebra (Fig. 3; also see Tornabene et al. 2016b, fig. 2). This is likely the case with *V.prometheus*, and we tentatively consider the vertebral counts to be 11+16 with a single anal-fin pterygiophore inserted before the haemal arch.

**Figure 5.**
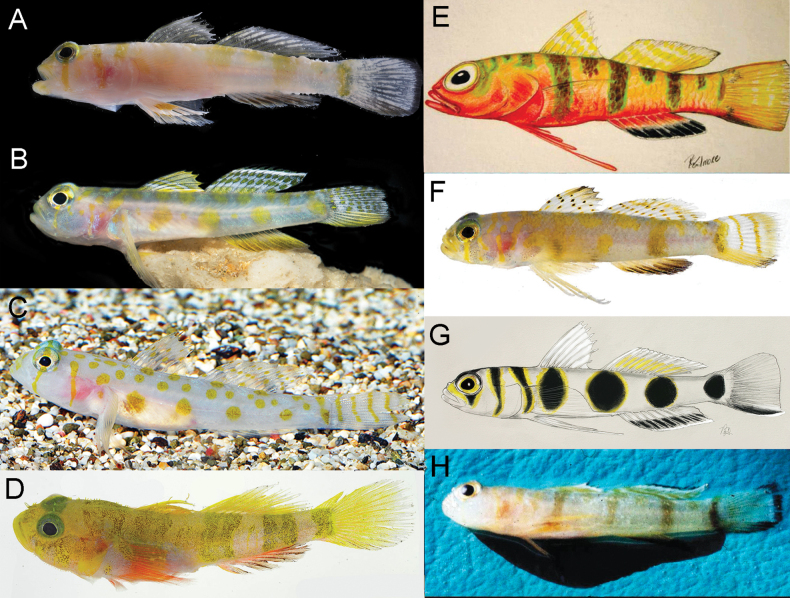
Live or fresh coloration of *Varicus* species (coloration unknown for *V.benthonis* and *V.bucca*) **A***V.adamsi*, photo by L. Tornabene **B***V.cephalocellatus*, photo by B. Brown **C***V.decorum*, photo by B. Brown **D***V.lacerta*, photo by C. Baldwin **E***V.marilynae*, illustration by R. G. Gilmore **F***V.veliguttatus*, photo by C. Baldwin **G***V.nigritus*, illustration by R. G. Gilmor **H***V.vespa*, photo by the crew of the R/V BELLOWS.

*Varicusprometheus* is easily distinguished from other species of *Varicus* and species in the morphologically similar genus *Psilotris* (Table 1) by the presence of four broad brown saddles on a uniformly yellow body. No other species of *Varicus* or *Psilotris* has such saddles, although a few species [e.g., *V.adamsi* Gilmore, Van Tassell & Tornabene, 2016; *V.vespa* (Hastings & Bortone, 1981); *V.marilynae* Gilmore, 1979] have complete or nearly complete vertical bars that are narrower than the saddles in *V.prometheus*, and *V.nigritus* has large dark blotches on the body that are as wide as the saddles in *V.prometheus* but are round rather than saddle shaped (Fig. 5). *Varicusprometheus* lacks scales on the trunk, whereas all other *Varicus* species except *V.lacerta* Tornabene, Robertson & Baldwin, 2016, and *V.decorum* Van Tassell, Baldwin & Tornabene, 2016, have many rows of scales on the trunk. Both *V.decorum* and *V.prometheus* possess modified basicaudal scales, which are lacking in *V.lacerta*. The unbranched pelvic rays of *V.prometheus* and the presence of one anal-fin pterygiophore (versus 2) inserted before the haemal arch distinguish it from all species of *Psilotris*.

**Table 1.** Important taxonomic characters for the genera *Varicus*, *Psilotris* and *Pinnichthys*.

Species	Second dorsal	Anal	Pectoral	AP	Pelvic rays 1–4	Papillae rows 5i/5s	Lateral scale rows	Basicaudal Scales
*Varicusprometheus* sp. nov	I,9	I,8	17	1	unbranched	connected	absent	present
*Varicusroatanensis* sp. nov	I,8	I,8	17	?	unbranched	?	28	present
*Varicusadamsi*	I,9	I,7–8	18	1	branched – fleshy pads	connected	21–24	present
*Varicusbenthonis*	I,8	I,7	16	1	branched	separate	12	present
*Varicusbucca*	I,9	I,7–8	16–19	1	unbranched or branched – fleshy pads	connected	27	present
*Varicuscephalocellatus*	I,10	I,9	19–20	1	unbranched	variable	12–23	present
*Varicusdecorum*	I,9	I,7–8	17	1	unbranched	connected	absent	present
*Varicuslacerta*	I,9	I,7	18	s	branched – feathery	connected	absent	absent
*Varicusmarilynae*	I,8	I,7	16–18	1	branched – fleshy pads	connected	18–19	present
*Varicusnigritus*	I,9	I,8	17	1	unbranched	connected	present (exact count unknown)	present
*Varicusveliguttatus*	I,8	I,6–7	17–19	1	unbranched	connected	23–34	present
*Varicusvespa*	I,9	I,7 (rarely I,6 or I,8)	15–17	1	branched – fleshy pads	separate	9–14	present
*Psilotrisalepis*	I,9 (rarely I,8)	I,7–8	15	2	branched	separate	absent	absent
*Psilotrisboehlkei*	I,9–10	I,9	16–18	2	branched	separate	absent	absent
*Psilotriscelsa*	I,9–10 (rarely I,8)	I,9–10 (rarely I,8)	16–17	2	branched	connected	absent	absent
*Psilotriskaufmani*	I,10 (rarely I,9)	I,10 (rarely I,9)	16–19	2	branched	connected	absent	absent
*Psilotrislaetarii*	I,9–10	I,7–8	15–17	2	branched	connected	absent	absent
*Psilotrislaurae*	I,9	I,8	18	1	branched	connected	absent	absent
*Psilotrisvantasselli*	I,9	I,8	15	1	branched	connected	absent	present
*Pinnichthysaimoriensis*	I,10–11	I,10	18–19	2	branched	connected	40–47	present
*Pinnichthysbilix*	I,11	I,10–11	19–20	2	branched	connected	30–35	present
*Pinnichthysprolata*	I,10–11	I,10–11	17–19	2	branched	connected	34–37	present
*Pinnichthyssaurimimica*	I,11	I,11	20	2	branched	connected	47–53	present
*Pinnichthysatrimela* (Pacific)	I,11	I,10	20	2	branched	connected	41	present

#### ﻿ Varicus roatanensis sp. nov.

Taxon classificationAnimaliaPerciformesGobiidae

EC48138A-17C1-5C7D-B542-33391B303D94

https://zoobank.org/D59D4338-07EB-4437-9607-F746940EE982

**Figure 6.**
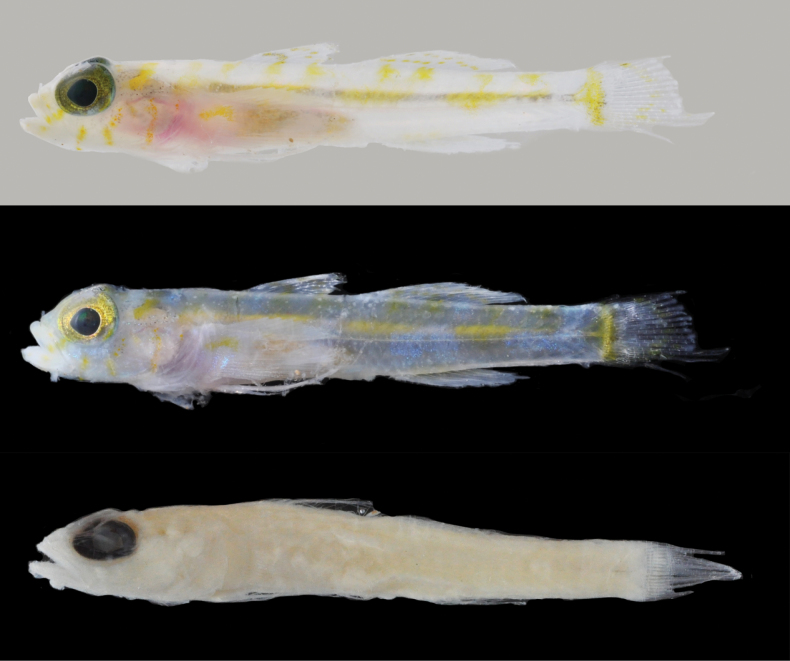
*Varicusroatanensis* sp. nov. holotype, UW158127, 18.5 mm SL, female, prior to preservation (top two photos) and preserved (bottom). Photos by L. Tornabene.

Fig. 6 Roatan goby, Gobio de Roatan (Spanish)

##### Type locality.

Roatan, Honduras; western Caribbean.

##### Holotype.

Honduras • 1 female 18.5 mm SL; western Caribbean, island of Roatan, west End, off Half Moon Bay, sta. IDABEL18-03; 16.304°N, 86.598°W; 237 m depth; 6 June 2018; Luke Tornabene, Rachel Manning, and Karl Stanley; 5% quinaldine-sulfate dispersed from Idabel submersible; UW 158127, DNA sample ROA18043.

##### Generic placement.

In addition to the molecular characters supporting the phylogenetic placement of this species, the following morphological characters support its inclusion in the genus *Varicus*: first dorsal spines VII; vertebrae 11+16; dorsal pterygiophore formula 3-221110; anal-fin rays I,9 or fewer (I,8 in *V.roatanensis*); head pores absent; pelvic fins completely separate, lacking both anterior frenum and membrane connecting bases of innermost pelvic-fin rays; fifth pelvic-fin ray unbranched.

##### Diagnosis.

*Varicusroatanensis* is distinguishable from all other *Varicus* species by the following combination of characters: second dorsal fin I,8; anal fin I,8; pectoral fin 17; body scaled with 28 lateral rows of ctenoid scales, modified basicaudal scales present; pelvic rays 1–4 unbranched; body white with a series of elongate yellow dashes along lateral midline with scattered yellow spots between lateral midline and dorsal midline; dorsal and caudal fins white with bright yellow spots in life, pectoral, pelvic, and anal fins white.

##### Description.

General shape: body widest and deepest at the head, trunk tapering in width and depth posteriorly, dorsal head profile moderately sloping from dorsum to tip of snout.

***Morphometrics (%SL)***: head length 30.3; eye diameter 10.8; snout length 5.9; upper-jaw length 10.3; post-orbital length 13.5; predorsal length 40.0; body depth at 1^st^ dorsal-fin origin 15.1; body depth at anal-fin origin 12.4; preanal length 54.1; body depth at caudal peduncle 9.2; caudal-peduncle length 9.2; pectoral-fin length 20.5; pelvic-fin length 25.9.

***Median and paired fins***: first dorsal fin VII; second dorsal fin I,8; anal fin I,8; pectoral fin 17; pelvic fins I,5, fins well separated, lacking both anterior frenum and membrane connecting bases of innermost rays; 4^th^ pelvic-fin ray longest, extending posteriorly to anus; rays 1–4 connected by a thin membrane, all unbranched without fleshy tips; 5^th^ ray unbranched and approximately 25% the length of the 4^th^ ray; caudal fin damaged, unable to determine fin-shape, branched caudal-fin rays13, segmented caudal-fin rays 17.

***Vertebral skeleton***: 27 vertebrae, 11 precaudal, and 16 caudal; dorsal pterygiophore formula 3-221110, unknown number of anal-fin pterygiophores inserted anterior to 1^st^ haemal spine, as radiographs were unclear.

***Head***: jaw terminal, angled approximately 50 degrees from the horizontal axis of body, extending posteriorly to vertical anterior end of pupil; anterior naris on elongate tube, posterior naris barely visible as an opening on a slightly raised rim immediately anterior to eye; eyes large, dorsolateral, extending slightly above head profile; narrow interorbital space, operculum opening slightly larger than pectoral fin base; condition and presence/absence of teeth undeterminable due to condition of specimen.

***Sensory papillae***: skin of holotype is heavily abraded and no papillae are visible on the head or body.

***Squamation***: trunk covered with ctenoid scales, extending anteriorly to pectoral-fin base; 28 scales in lateral series, approximately 9 or 10 transverse scale rows; no scales on head; 2 modified basicaudal scales with enlarged ctenii present at dorsal and central margins at base of caudal-fin.

***Genitalia***: female with short, rounded papilla, wider at base and tapering to a slightly conical point; male unknown.

***Color before preservation* (Fig. 6)**: body translucent white with pale to bright yellow markings which are more visible when fish was photographed against white vs. black background. Head white with three slender bright yellow stripes below and posterior to eye, a slender yellow bar running down the center of the operculum; and with scattered melanophores, silver iridiophores, and bright yellow xanthrophores extending posterior from eye onto opercle; iris gold, heavily speckled with green-yellow dots; a thin silvery-white inner ring around pupil; three indistinct yellow bands across the nape, last one at level of origin of first dorsal fin. Body translucent white with three elongate yellow dashes along lateral midline, first dash under first dorsal fin, second dash below origin of second dorsal fin, third dash positioned below posterior half of second dorsal fin; nine small yellow spots extending ventrally from dorsal midline, first spot starting on nape, last spot above middle of caudal peduncle. First and second dorsal fins pale, each interradial membrane with one or two bright yellow spots, giving faint impression of one or two yellow stripes across fins. Caudal fin pale with a faint yellow curved bar across middle of rays, and a bright yellow vertical bar across base of caudal fin, with a triangular extension onto caudal peduncle. Anal fin pale, slightly translucent. Pectoral-fin base white with small horizontal patch of yellow on the dorsal part of fin base; pectoral rays white and semitransparent; pelvic fins pale.

***Color in preservation* (Fig. 6)**: body uniformly pale yellow with no visible pigmentation on head, trunk or fins.

##### Habitat.

Collected at 237 m. Specimen found on a silt-covered boulder with small 3–10 cm diameter caves and crevices.

##### Distribution.

Known only from the type locality off Roatan, Honduras.

##### Etymology.

The specific epithet *roatanensis* refers to the type locality, Roatan, Honduras.

##### Remarks

**(Table 1).***Varicusroatanensis* can be distinguished from *V.decorum*, *V.lacerta* and *V.prometheus* and all species of *Psilotris* by the presence of multiple rows of scales on the side of the body (versus body scales absent; two modified basicaudal scales present in *V.decorum*, *V.prometheus*, and *P.vantasselli*). The unbranched pelvic-fin rays in *V.roatanensis* distinguish it from *V.adamsi*, *V.benthonis* (Ginsburg, 1943), *V.marilynae* and *V.vespa*, all of which have rays 1–4 branched (*V.adamsi*, *V.marilynae* and *V.vespa* also with fleshy pads at the tips of rays). *Varicusroatanensis* is most similar to *V.bucca* Robins & Böhlke, 1961, *V.cephalocellatus* Gilmore, Van Tassell & Baldwin, 2016, *V.nigritus* Gilmore, Van Tassell & Baldwin, 2016, and *V.veliguttatus* Van Tassell, Baldwin & Gilmore, 2016, in having scales on the body and unbranched pelvic-fin rays but differs from each of these species in body coloration. *Varicusroatanensis* is the only species in the genus to have elongate yellow dashes along the lateral midline, whereas *V.veliguttatus*, *V.nigritus* and *V.decorum* have distinct yellow or dark round blotches, saddles, or vertical bars on the body. While the fresh or living coloration of *V.bucca* is not known, and the type series may represent several species (Tornabene et al. 2016b), the description and illustration of the preserved holotype clearly indicate a very dark anal fin that is heavily covered with melanophores (Robins and Böhlke 1961). This pattern is shared with several other species of *Varicus* (e.g., *V.adamsi*, *V.marilynae*, *V.nigritus*, *V.vespa*), but differs from that of *V.roatanensis*, which has a pale anal fin with a very faint yellow shading near the bases of the rays.

#### ﻿ Pinnichthys aimoriensis

Taxon classificationAnimaliaGobiiformesGobiidae

Van Tassell & Tornabene, 2016

9E60D061-3004-5CA4-8EA3-6A5FF65FE5A1

**Figure 7.**
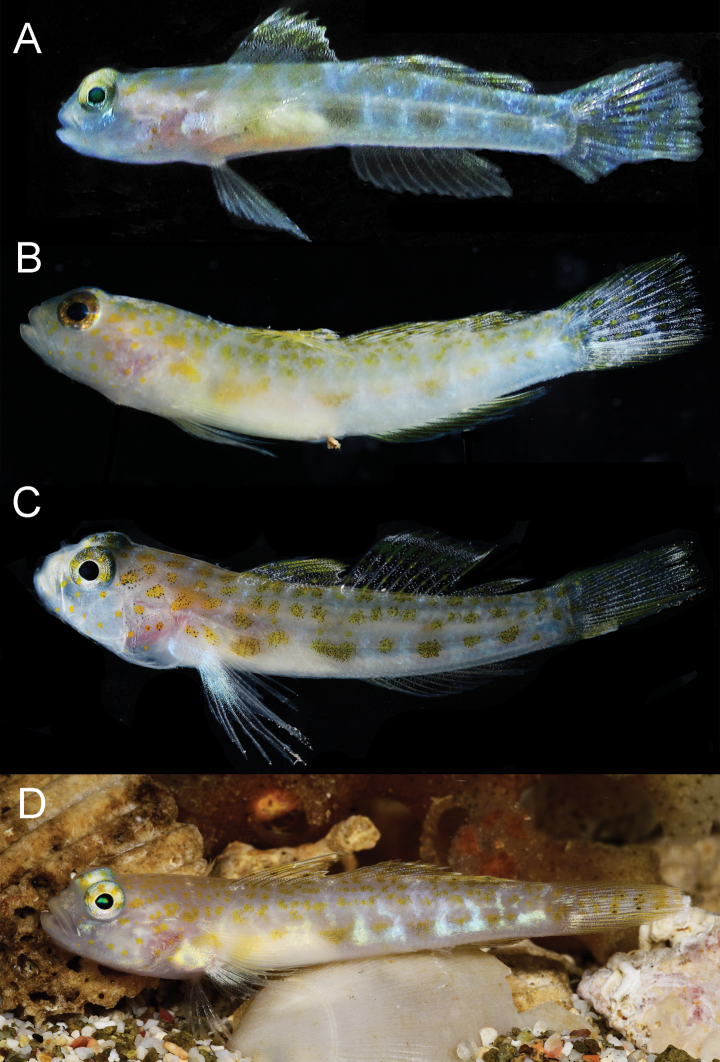
*Pinnichthysaimoriensis* live or fresh coloration on dark backgrounds **A** CIUFES 2414, holotype, Brazil **B** USNM 442071, Bonaire **C** USNM 442696, St. Eustatius **D** USNM 442696, live, St. Eustatius. Photos by C. Baldwin (**A–C**) and B. Brown (**D**).

**Figure 8.**
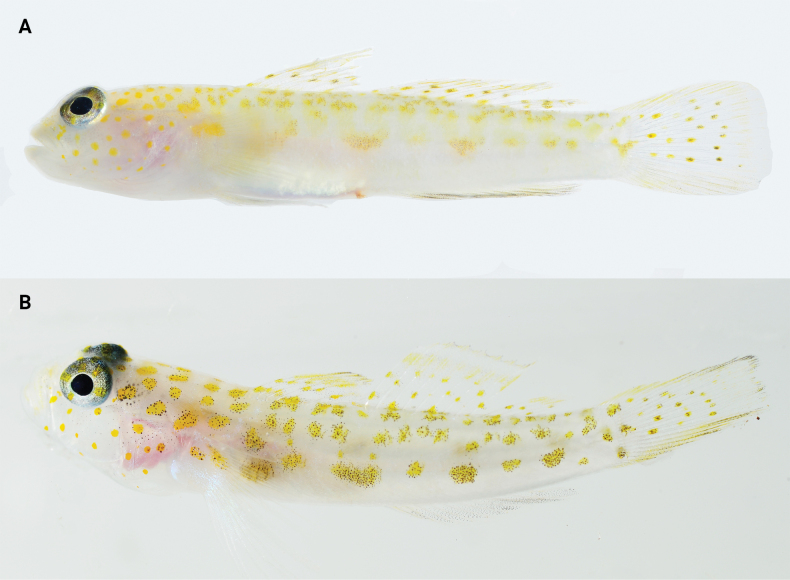
*Pinnichthysaimoriensis* fresh coloration on light background **A** USNM 442071, Bonaire **B** USNM 442696, St. Eustatius. Photos by C. Baldwin.

Figs 7 , 8 Thiony’s Goby

##### New material examined.

Sint Eustatius • 1 male 19.5 mm SL; eastern Caribbean, SW side of island, Kay Bay, South and Southeast of R/V Chapman Mooring, sta. CURASUB17-17; 17.4600°N, 62.9816°W; 96.3 m depth; 15 April 2017; C. Baldwin, L. Tornabene, B. Brandt, and J. Casey; quinaldine dispersed from Curasub submersible; USNM 442696, DNA sample EUS17043. BONAIRE • 1 female 28.6 mm SL; southern Caribbean, Belnem, South of Punt Vierkant, sta. CURASUB17-08; 12.095°N, 68.2966°W; 162–164 m depth; 17 January 2017; C. Baldwin, L. Tornabene, B. Brandt, T. Devine; quinaldine dispersed from Curasub; USNM 442071, DNA sample BON17071.

Data from the two additional specimens examined expand upon the known morphological variation within *Pinnichthysaimoriensis* (Table 2). We provide an updated diagnosis for the species and a description of fresh coloration of the two new specimens (new information in **bold**).

**Table 2.** Comparison of new data for *Pinnichthysaimoriensis* and data from the type series. Values for new specimens that are outside the range of the type series are in bold.

	USNM 442696	USNM 442071	CIUFES 2414 (holotype)	All types
SL	19.5	28.6	22.4	16.4–22.4
Sex	male	female	male	2 males, 1 female
Morphometrics in % SL				
Eye diameter	9.2	8.4	8.7	8.6–9.15
Jaw length	9.7	10.1	9.6	9.6–11.5
Snout length	6.2	5.6	5.8	5.2–7.0
Head length	23.6	29.7	27.9	27.9–29.5
Postorbital length	**10.3**	14	13.5	13.5–16.6
Depth at first dorsal-fin origin	17.4	18.5	16.3	15.8–16.5
Depth at anal-fin origin	17.4	17.1	17.3	14.6–17.3
Least caudal peduncle depth	**9.7**	11.2	12.5	10.4–12.5
Caudal peduncle length	**15.4**	17.5	22.9	18.8–23.0
Caudal-fin length	23.4	22.21	26.3	26.3–27.8
Pectoral-fin length	**16.8**	26.2	20.9	19.8–25.0
First dorsal-fin elements	VII	VII	VII	VII
Second dorsal-fin elements	**I,11**	**I,11**	I,10	I,10
Anal-fin elements	I,10	I,10	I,10	I,10
Pectoral-fin rays	19	19	18/19	18–19
Caudal-fin rays segmented, branched	17, **15**	17,14	17;14	17;14
Pelvic-fin elements	I,V	I,V	I,V	I,V
Length of fifth pelvic-fin ray relative to fourth	**3/4**	1/2	1/2	1/2
Vertebrae – precaudal + caudal	?	11+16	11+16	11+16
Dorsal-fin pterygiophore pattern	?	3-221110	3-221110	3-221110
Anal-fin pterygiophores inserted before haemal arch	?	2	2	2
Pelvic fins 1–4	branched, no fleshy tips	branched, no fleshy tips	branched, no fleshy tips	branched, no fleshy tips
Lateral scale rows	43	47	40–45*	40–47
Tranverse scale rows	11	14	10**	10–13**

* some scales missing anteriorly, scale rows estimated from scale pockets. ** holotype 10, paratypes 11–13, counts originally described as 8–9.

##### Diagnosis.

Side of body with 40–47 scale rows extending anteriorly to pectoral base; modified basicaudal scales present; first dorsal fin VII, without notably elongate spines, **second dorsal I,10–11**; anal fin I,10, rays fork only once near tips; pelvic fins well separated, no anterior frenum and no membrane connecting base of innermost rays; **fifth pelvic-fin ray one half to three quarters** the length of the fourth and unbranched; pelvic-fin rays 1–4 branched, without fleshy tips; papillae rows 5s and 5i separate, lacking a papilla that would result in their forming a single continuous transverse row; interorbital papillae row pb’, pc’, and pe’ present; head and preopercle canals and pores absent; two anal-fin pterygiophores inserted anterior to haemal arch.

***Color before preservation* (Figs 7, 8)**: Background color of body, head and fins pale to translucent; eye with five to six yellow spots spaced evenly around iris, iris silvery white with slight iridescent green tint; side of head and nape with numerous distinct small yellow spots; paired yellow spots on upper side of nape continue posteriorly along each side of the dorsal midline and extend ventrally onto upper portion of trunk in approximately two irregularly rows of yellow spots ending on upper portion of caudal peduncle; body with four round to slightly horizontally elongate yellow to yellowish-brown blotches along lateral midline, anteriormost botch largest, approximately equal to eye diameter, located beneath first dorsal fin; three smaller yellow to yellowish-brown spots on lateral midline, each located between larger blotches; both dorsal fins and caudal fins speckled with minute iridiophores; base of first dorsal fin pale, middle portion of fin bright yellow, distal margin of fin white; second dorsal fin with numerous yellow spots on rays; caudal fin sometimes with narrow yellow stripe on dorsal and ventral margins, rest of fin with numerous yellow spots, loosely arranged into three to four vertical rows, yellow spots on head and body sometimes with dark centers or margins of melanophores; base of anal fin pale to yellow, outer half of fin heavily covered with melanophores giving a uniformly dusky to black appearance; pectoral-fin base and rays pale with one or two small yellow blotches on dorsal half of pectoral-fin base and origin of dorsal-most rays; pelvic fins pale to faintly yellow.

##### Habitat.

The type series off Espírito Santo, Brazil was collected near the Peroá natural gas platform at 70 m depth on a substrate of rhodoliths and other calcareous substrate. The Bonaire specimen was collected at 164 m on a moderately steep slope with short rock ledges, small caves and crevices – all of which were covered with fine sand. It was collected alongside two specimens of *Varicusdecorum*. The St. Eustatius specimen was collected on a sand and *Halimeda* rubble substrate in close proximity to an *Ircinia* sp. sponge and several ~1 m diameter boulders covered with encrusting algae and sponges.

##### Distribution.

**Figure 9.**
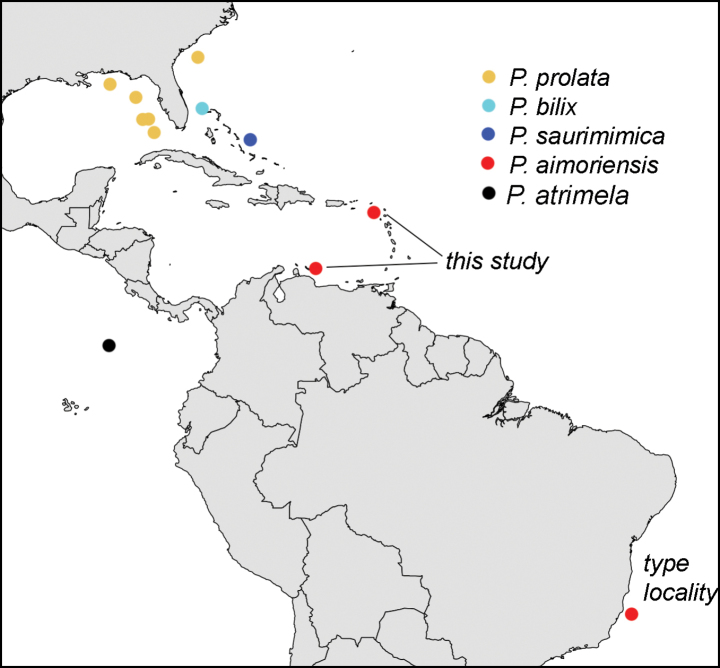
Distribution of *Pinnichthys* based on all known specimens.

Known from the margin of the continental shelf of Brazil off Espírito Santo, in the eastern Caribbean off Sint Eustatius, and in the southern Caribbean off Bonaire (Fig. 9).

##### Remarks.

**Figure 10.**
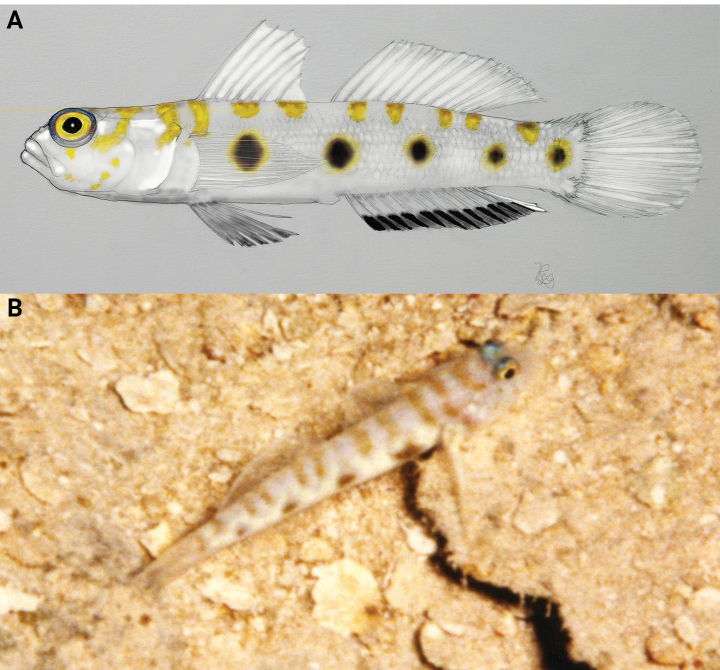
Live or fresh coloration of *Pinnichthyssaurimimica***A** illustration by R. G. Gilmore **B** photograph by R.G. Gilmore from the Johnson Sea Link II submersible.

The data presented here for the two Caribbean specimens of *Pinnichthysaimoriensis* increase the range of several morphological characters, including the number of elements in the second dorsal fin (was I,10, now I,10–11). *Pinnichthysaimoriensis* can be distinguished from *P.bilix* (Hastings & Findley, 2013) and *P.prolata* (Hastings & Findley, 2015) in having more lateral scale rows (40–47 vs 30–37). *Pinnichthysaimoriensis* lacks the elongate dorsal spines that are present in *P.bilix*, and lacks the elongate fifth pelvic ray present in *P.prolata* (fifth pelvic ray falling well short of anus when adpressed, versus reaching anus or beyond in *P.prolata*). *Pinnichthysaimoriensis* can be futher distinguished from *P.saurimimica* Gilmore, Van Tassell & Tornabene, 2016, in having fewer lateral scale rows (40–47 vs. 47–53) and fewer pectoral-fin rays (18–19 vs 20), and in live coloration (Figs 7, 8, 10). *Pinnichthyssaurimimica* lacks the smaller spots on the body located between the larger blotches along the lateral midline (Fig. 10), which are present in *P.aimoriensis* (Figs 7, 8). While *P.aimoriensis* has many very small spots along the nape and dorsal midline arranged into roughly two irregular rows continuing down each side of the dorsal surface of the trunk (Fig. 7), the pattern in *P.saurimimica* is that of ~10 narrow short saddles or evenly-spaced vertically elongate spots along the nape and dorsal midline (Fig. 10).

##### Remark on *Pinnichthysatrimela*.

Tornabene et al. (2016b, pg. 14) erected the genus *Pinnichthys* for five species, including the eastern Pacific species *P.atrimela* (Bussing, 1997) (formerly *Chriolepisatrimelum*). However, in their table 2, they incorrectly listed the new classification of this species as *Chriolepisatrimela*, forgetting to change the genus from *Chriolepis* to *Pinnichthys*. Table 1 here now correctly lists all members of *Pinnichthys*, including *P.atrimela*.

## Supplementary Material

XML Treatment for Varicus prometheus

XML Treatment for Varicus roatanensis

XML Treatment for Pinnichthys aimoriensis

## ﻿Additional information

### Author ORCIDs

Carole C. Baldwin https://orcid.org/0000-0002-2875-0474

D. Ross Robertson https://orcid.org/0000-0003-3972-149X

Claudia C. Lardizábal https://orcid.org/0000-0002-2802-7593

Luke Tornabene https://orcid.org/0000-0002-0673-2320

### Data availability

All of the data that support the findings of this study are available in the main text or Supplementary Information.

## ﻿Appendix 1

**Table A1.** GenBank accession numbers for new sequences.

Species	Catalog number	GenBank cytb	GenBank rag1	GenBank sreb2	GenBank zic1
*Pinnichthysaimoriensis*	USNM 442696	OR437371	OR437374	NA	NA
*Pinnichthysaimoriensis*	USNM 442071	OR437372	OR437375	OR437377	NA
*Varicusprometheus*	UW 158119	OR437370	NA	NA	OR437379
*Varicusroatanensis*	UW 158127	OR437369	OR437373	OR437376	OR437378

## Supplementary materials

Supplementary material 1 Maximum clade credibility tree from Bayesian analysis with all samples Data type tre Explanation note The maximum clade credibility tree summarized from post-burnin trees of the MrBayes analysis. https://binary.pensoft.net/file/908656This dataset is made available under the Open Database License (http://opendatacommons.org/licenses/odbl/1.0/). The Open Database License (ODbL) is a license agreement intended to allow users to freely share, modify, and use this Dataset while maintaining this same freedom for others, provided that the original source and author(s) are credited.Luke Tornabene, Katlyn M. Fuentes
